# Green Alternatives to Synthetic Antioxidants, Antimicrobials, Nitrates, and Nitrites in Clean Label Spanish Chorizo

**DOI:** 10.3390/antiox8060184

**Published:** 2019-06-19

**Authors:** Lorena Martínez, Pedro Bastida, Julian Castillo, Gaspar Ros, Gema Nieto

**Affiliations:** 1 Department of Food Technology, Nutrition and Food Science, Veterinary Faculty University of Murcia, Campus de Espinardo, 30100 Espinardo, Murcia, Spain; lorena.martinez23@um.es (L.M.); pebastidaaz@gmail.com (P.B.); gros@um.es (G.R.); 2 Research and Development Department of Nutrafur-Frutarom Group, Camino Viejo de Pliego s/n, 30820 Alcantarilla, Murcia, Spain; j.castillo@Nutrafur.com

**Keywords:** antioxidant, antimicrobial, phenolic compounds, nitrate, chorizo, *Clostridium perfringens*, volatile compounds

## Abstract

Natural extracts obtained from fruit and vegetable processing are important sources of phenolic compounds and nitrates, with excellent antioxidant and antimicrobial properties. The aim of this study was to characterize and determine the antioxidant and antimicrobial capacity of several natural extracts (citric (Ct), acerola (Ac), rosemary (R), paprika, garlic, oregano, beet (B), lettuce (L), arugula (A), spinach (S), chard (Ch), celery (Ce), and watercress (W)), both in vitro and applied to a cured meat product (chorizo). For that, the volatile compounds by GC-MS and microbial growth were determined. The total phenolic and nitrate contents were measured and related with their antioxidant capacity (measured by DPPH, ABTS, FRAP, and ORAC methods) and antimicrobial capacity against *Clostridium perfringens* growth in vitro. In order to study the antioxidant and antimicrobial activities of the extracts in food, their properties were also measured in Spanish chorizo enriched with these natural extracts. R and Ct showed the highest antioxidant capacity, however, natural nitrate sources (B, L, A, S, Ch, Ce, and W) also presented excellent antimicrobial activity against *C. perfringens*. The incorporation of these extracts as preservatives in Spanish chorizo also presented excellent antioxidant and antimicrobial capacities and could be an excellent strategy in order to produce clean label dry-cured meat products.

## 1. Introduction

One of the most important difficulties faced in the food industry is the great quantity of waste generated from fruit and vegetable processing, which has become a problem for the environment and is a large capital investment for companies. However, residues, such as peels, seeds, or leaves, are rich in phenolic compounds and other bioactive substances, such as nitrates, with potential antioxidant and antimicrobial properties that could substitute synthetic additives in the manufacturing of new and functional food products with healthier benefits for the human body [1].

Antioxidant compounds are substances that retard the oxidation of food products by inhibiting free radical formation or interrupting this pathway through some specific mechanisms. One of these pathways is hydrogen atom transference, during which an antioxidant compound (AH) gives an H to a free radical ($R^{⏹}$), generating a more stable radical $\left(A^{⏹}\right)$ $\left(R^{⏹}+\;\mathrm{AH}\;\to \;\mathrm{RH}+A^{⏹}\right)$. The other pathway is electron transference, in which AH gives an electron in order to reduce the free radical $\left(R^{⏹}+\mathrm{AH}\to {\;R}^{-}+{\mathrm{AH}}^{⏹}\right)$ [1]. In parallel, regarding their chemical nature and origin, these compounds could also prevent against bacterial development through the inhibition of several functions, such as bacteria cell wall maintenance, protein synthesis, transport, or DNA replication, as principal antimicrobial mechanisms of action [2]. On the other hand, nitrate and nitrite salts are used in food products for the control and prevention of *C. Botullinum* growth. However, the consumption of this additive is regulated because this substance is naturally present in soil, vegetables, water, and animals, and normal levels have increased in recent years due to the use of nitrogen fertilizers. For this reason, the use of natural sources of nitrates from green leaf vegetables could prevent the abuse of synthetic nitrates in the development of clean label food products [3].

Consequently, in this current study, several extracts with antioxidant and antimicrobial properties (rosemary, oregano, citrus extract, and acerola), spices (paprika), natural sources of nitrates (beet (B), lettuce (L), arugula (A), spinach (S), chard (Ch), celery (Ce), and watercress), and traditional ingredients (garlic) added to meat products were characterized. Rosemary (*Rosmarinus officinalis,* L.) is a natural woody perennial green herb from the Mediterranean region that is rich in vitamins A, C, B1, B6, and B9; minerals, such as Mg, Ca, Cu, Fe, and Mn; as well as phenolic compounds, such as rosmarinic acid and diterpenes (carnosic acid, carnosol, among others). Regular consumption of this herb has shown many beneficial effects in human health [4], acting as an antioxidant and antibacterial agent [5]. Citric extracts, obtained from a mix of sweet orange (*Citrus sinensis*) and bitter orange (*Citrus aurantium*), are rich in bioactive compounds, such as naringin and hesperidin, and are both glycosides of flavanones that act as antioxidants due to their great ability to chelate iron and their activity of sweeping their hydroxyl groups [6]. In addition, acerola, also known as *Malpiguia emarginata*, is a plant native to Central and South America and is one of the most important sources of vitamin C (ascorbic acid), along with carotenoids and bioflavonoids as anthocyanins and flavonols that increase its antioxidant power [7].

Other ingredients traditionally added to meat products, such as chorizo, with potential functionality have been studied. Firstly, paprika is a red powder condiment made from red peppers, *Capsicum annum*. It is one of the most commonly used species as a natural colorant in the preparation of cured sausages due to its characteristic aroma, colour, flavor, and antioxidant power. In addition, paprika is an important source of bioactive compounds, such as carotenoids (β-carotene and β-cryptoxanthin as the major), vitamin E and C, and phenolic compounds (feruloyl glycosides, luteolin, and quercetin glycosides) with excellent antimicrobial and antioxidant properties, among other health benefits [8,9,10]. Sencondly, garlic (*Allium sativum*) has been studied, emphasizing its antioxidant and antimicrobial properties, which have been associated with the high concentration of allicin and other organosulfuric compounds, such as thiosulfinates, and phenolic compounds, such as flavonoids and pherulic acids [11]. Likewise, oregano (*Origanum vulgare*) is also commonly used as a flavouring in cured or fresh meat products with excellent antibiotic and antioxidant properties related to its principal components, such as rosmarinic acid and monoterpenes, carvacrol, and thymol, among other phenolic compounds, such as γ-terpinene and *p*-cymene [12].

Finally, natural nitrate sources have been studied in order to find potential substitutes of synthetic nitrates and nitrites used in dry-cured food products, such as green leaf vegetables, rich in nitrates (beet, lettuce, arugula, watercress, celery, spinach, and chard) [13,14]. The addition of synthetic nitrites (E-249 and E-250) to this kind of meat product acts as an antioxidant against lipid oxidation, antimicrobial against anaerobic bacteria growth, and colour preservative. However, regular consumption of nitrites can affect the human body in different ways, such as causing allergic problems and reacting with hemoglobin to produce methemoglobin in the blood (reducing the transport capacity of oxygen). When cyanosis occurs, due to the high methemoglobin content, it results in symptoms of low oxygen level, weakness, dyspnea, headache, tachycardia, etc. [15]. Moreover, nitrites can also react with amine/amide to produce nitrosamine, which have carcinogenic properties, and their consumption can increase gastrointestinal cancer risk [15]. Regarding nitrate, the incorporation of synthetic nitrate in cured meat products can produce a similar effect, because nitrate is gradually reduced to nitrite in meat, unless it is in a minor concentration that the human body can metabolize and eliminate before being affected. Consequently, nitrate addition is a better option for the maintenance of cured meat products’ quality. In addition, in order to adapt our product to the new trends of clean label meat product elaboration, synthetic additives have been substituted by natural nitrate sources to create a Spanish chorizo that is free of them.

The main objective of the present study was comparatively test natural antioxidant extracts together with the traditional ingredients of cured meat products and natural sources of nitrates obtained from green leaf vegetables for their potential use in the food industry as antioxidant and antimicrobial extracts. For this reason, the antioxidant and antimicrobial capacities of each extract were evaluated, in relation to their total phenolic and nitrate content, and tested in a dry-cured meat product: Spanish chorizo.

## 2. Materials and Methods

### 2.1. Plant, Spices, and Vegetable Extracts

Natural antioxidant extracts were obtained from the non-edible part of citrics (*Citrus sinensis* L.) (Ct), with 55.11% hesperidin, and rosemary (*Rosmarinus officinalis* L.) (R), with 14.59% carnosic acid, 5.84% carnosol, and 0.60% 12-O-methylcarnosic acid. Both were supplied by Nutrafur-Frutarom, S. A. (Alcantarilla, Murcia, Spain). Acerola extract (*Malpighia emarginata*) (Ac), with 5% vitamin C, was supplied by Ferrer Alimentación, S. A. (Barcelona, Spain), and it was extracted from this fruit.

Paprika, garlic, and oregano powder as well as beet (B), lettuce (L), arugula (A), spinach (S), celery (Ce), chard (Ch), and watercress (W) were bought in a local supermarket (Hipercor, S. A., El Tiro, Murcia, Spain). In total, 500 g of the edible part of each vegetable were chopped and mixed with 150 mL of distilled water in a Thermomix^®^. Each mix was frozen for 48 h at −80 °C and lyophilized in a freezing dryer Telstar, model Cryodos-80 (Telstar, Terrasa, Spain).

#### 2.1.1. Total Phenolic Content (TPC)

The total phenolic content (TPC) was determined quantitatively using the Folin–Ciocalteu reagent and gallic acid as the standard [14]. Each extract was diluted with water or ethanol, according to its polarity, in a 1000 ppm solution. Then, 100 µL of sample or standard solution of gallic acid at different concentrations (20, 40, 60, 80, and 100 mg per L) were mixed with 500 µL of 0.2 N Folin–Ciocalteau phenol reagent and 400 µL of 2% Na_2_CO_3_ in plastic cuvettes. After incubation for 1 h, the absorbance was measured at 750 nm. The TPC is expressed as mg gallic acid equivalents (GAEs) per g of extract. Results were compared with the USDA Database for the Total Phenolic Content (TPC) in order to have a comparative reference of the obtained results.

#### 2.1.2. Total Nitrate Content (TNC)

The total nitrate content (TNC) of natural extracts obtained from green leaf vegetables was evaluated following the method described by Cataldo et al. [15]. For that, 0.5 g of extract were weighted and diluted with 50 mL of destillated water. Samples were placed in a 100 °C water bath for 30 min and filtered in order to extract the N-NO_3_. Aliquots of 200 µL were added to two different glass tubes (one sample and one blank). In total, 800 µL of 5% salicylic acid (*w*/*v*) in sulfuric acid (SA-H_2_SO_4_) were added to the sample tube and 800 µL of sulfuric acid were added to the blank tube. Tubes were left to react for 20 min and then 19 mL of 2N NaOH were added. Then, tubes were incubated at room temperature in the dark for 24 h. After this time, the absorbance of each sample was measured at 410 nm. A standard curve of KNO_3_ at different concentrations was elaborated following the same method. The obtained results are expressed in ppm NO_3_^−^.

#### 2.1.3. Antioxidant Activity

The antioxidant activity related with the chelating power to different free radicals (%) was firstly measured using the 2,2-diphenyl-1-picrylhydrazyl (DPPH) free radical scavening method, described by Brand-Williams et al. [16] and Sánchez-Moreno et al. [17]. Each extract was diluted in a 1000 ppm solution with water or ethanol. A DPPH radical solution was prepared with 0.0063 g of DPPH in 250 mL of ethanol (99%). Then, 3.9 mL of DPPH radical solution was added to 100 µL of sample. After incubation in the dark at room temperature for 30 min, the absorbance was measured at 515 nm. The chelating activity percent was calculated using the next formula: ((Abs DPPH (initial) − Abs DPPH (final “added sample”))/Abs DPPH (initial)) × 100.

Secondly, the radical cation scavenging capacity against ABTS+ radical was measured as by Re et al. [18]. A 1000 ppm solution of each extract was mixed with water or ethanol. ABTS radical cations were prepared by reacting 7 mM ABTS (2,2-Azinobis (3-ethylbenzothiazolin)-6-sulphonic acid) with 2.45 mM potassium persulphate (1:1, *v*/*v*), pH = 7.4. This solution was diluted with distilled water to an absorbance of 0.7000 at 734 nm. In total, 1 mL of ABTS solution was added to 100 µL of sample. After 2 min incubation, the absorbance was measured at 734 nm. The chelating activity percent was calculated using the next formula: ((Abs ABTS (initial) − Abs ABTS (final “added sample”))/Abs ABTS (initial)) × 100.

Otherwise, the hydrophilic antioxidant capacity was measured using the ORAC (Oxygen Radical Absorbance Capacity) method described by Prior et al. [19]. The reaction was carried out in a 0.075 M phosphate buffer (pH 7.0). For this, 20 µL of sample, at different concentrations, and Trolox standard solutions (6.25, 12.5, 25, 50 µM) were pipetted into the wells of a 96-well black microplate. When the microplate was ready, 200 µL of 0.04 µM fluorescein were dispensed into each well. Samples were incubated for 15 min at 37 °C in the dark and the reaction was started by adding 20 µL of 40 mM AAPH to each well. The fluorescence decay was measured every 2 min at 37 °C using the microplate reader, Biotek Synergy HT (Biotek Intruments, Winooski, VT, USA), at a 485 nm excitation and 538 nm emission until zero fluorescence was detected and a UV2 spectrophotometer (Pye Unicam Ltd, Cambridge, UK) at different wavelengths depending on the method to be performed. All samples were prepared in triplicate and at a mínimum of three different concentrations. The antioxidant activity of the sample is expressed as µM of Trolox equivalents (TE) per g of extract, with the following formula: (C × DF)/a, where *C* is obtained from the area under the fluorescence decay curve of fluorescein in the presence of the sample (AUC net = AUC sample – AUC blank); *DF* is the dilution factor; and *a* is the weight of the sample. 

The ferric ion reducing antioxidant power assay (FRAP) was also carried out following the method described by Benzie and Strain [20] with some modifications. Each extract was diluted in a 1000 ppm solution using water or ethanol. Then, the FRAP reagent was prepared with 20 mL of 300 mmol/L acetate buffer, pH = 3.6, 2 mL 20 mmol/L FeCl_3_ · 6 H_2_O, and 2 mL 10 mmol/L TPTZ (2,4,6-tripyridyl-s-triazine) in 40 mmol/L HCl. After, 1 mL of the FRAP reagent was mixed with 100 µL of sample or a standard solution of 500 µM Trolox in plastic cuvettes. After incubation for 4 min, the absorbance was measured at 593 nm. The antioxidant power was expressed as µM Trolox equivalents (TE) per g extract.

#### 2.1.4. Antimicrobial Activity

The method was carried out according to Charlebois et al. [21] with some modifications. Firstly, each extract was diluted until 1000, 500, and 250 ppm. In parallel, the *Clostridium perfringens* strain, NCTC 8237 CECT 376, was manipulated following the reference strain recommended by UNE-CEN ISO/TS 11133 and EN ISO 7937. Colonies incubated in a plate for 48 h at 37 °C under anaerobic conditions (AnaeroGen TM, ThermoFisher Scientific, Waltham, Massachusetts, USA) were resuspended until a concentration of 1.5 McFarland. Then, 50 µL of that solution were mixed with 50 µL of BHI (Brain Heart Infusion) broth and 50 µL of extract dilution in a 96-well microplate. Plates were incubated under anaerobic conditions at 37 °C and measurement at 600 nm of the optical density of the medium was carried out at time 0, 24, and 48 h of incubation using the Synergy HT plate reader (Biotek Instruments, Winooski, VT, USA). Results are expresed in CFU (colony forming unit).

### 2.2. Cured Meat Product: Spanish Chorizo

#### 2.2.1. Samples Preparation

Eight different batches (10 samples per batch) of Spanish chorizo were manufactured using the recipe shown in Table 1. Minced meat was purchased in a local supermarket, Hipercor, S.A. (Murcia, Spain). Dextrose, meat protein, and the commercial mix of additives and spices composed of spices, salt, dextrose, lactose, milk protein, emulsifiers (triphosphates E-451, diphosphate E-450), flavour enhancer (monosodium glutamate E-621), preservative (sodium nitrate E-251), antioxidant (sodium ascorbate E-301), and colouring (carminic acid E-120), was used as the control sample (C). A comercial starter culture composed of *Pediococcus* (50 g per 100 g culture), *Staphylococcus xylosus* (25 g per 100 g culture), and *Staphylococcus carnosus* (25 g per 100 g culture). The lyophilised cultures were rehydrated (50 g in 750 mL of chlorinated-free water) for 8 h and then sown in the mass at a rate of 6 × 10^7^ CFU/g. Traditional ingredients of Spanish chorizo (paprika, garlic powder, and oregano) were purchased in a local supermarket, Hipercor, S. A. (Murcia, Spain).

The meat was then mixed with the starter cultures, additives, spices, and natural extracts. The paste was stuffed into swine casing, slightly curved, 40 to 43 mm caliber, and 300 to 400 mm in length, using an automatic stuffer (Silvercrest^®^ kitchen tools, Barcelona, Spain). The casing was supplied by Tripas De Murcia, S.L. (Alhama de Murcia, Murcia, Spain) and was previously desalted and washed with chlorinated-free water. The Spanish chorizo samples were labelled and placed in an air-drying chamber, Binder 115 redLine RI (Tuttlingen, Germany), set at 22 ± 1 °C and 90 ± 5% R.H. for two days. After that, the temperature and humidity were adjusted to 14 ± 1 °C and 70 ± 5% R.H. for 20 days. Analysis were carried out at 0 and 50 days. After the curation process, to study the shelf life, the Spanish chorizo samples were vacuum packaged and then stored at 5 ± 1 °C and 65 ± 5% R.H. for 125 days. Analyses were carried out at days 0, 25, 50, 75, and 125 from elaboration. Microbiological growth was determined at day 50, while volatile compounds were measured at days 0, 25, 75, and 125.

#### 2.2.2. Volatile Compounds by GC-MC

Lipid oxidation was related to the concentration of volatile compounds, following the method described by Lorenzo, Bedia, and Bañón [22] with some modifications. For that, 5 g of Spanish chorizo sample were placed in glass vials. Volatile compounds were extracted using solid-phase microextraction (SPME). An SPME device (Supelco, Bellefonte, PA, USA) containing a fused-silica fibre (10 mm length) (polydimethylsiloxane/divinylbenzene (PDMS/DVB)) was used. Extraction was performed at 35 °C for 30 min. Once sampling was finished, the fibre was withdrawn into the needle and transferred to the injection port of the gas chromatograph-mass spectrometer (GC-MS) system. Volatile compounds were analysed in duplicate in all samples at days 0, 25, 75, and 125 from elaboration. Analyses were performed on a Hewlett-Packard 6890 N Series GC gas chromatograph fitted with an HP 5973 mass spectrometer and an MSD Chemstation (Hewlett–Packard, Palo Alto, CA, USA). A split injection port was used to thermally desorb the volatiles from the SPME fibre onto the front of the DB-624 capillary column (J&W scientific: 30 m × 0.25 mm id, 1.4 µm film thickness). Helium was used as a carrier gas with a linear velocity of 36 cm/s. The temperature programme was 40 °C for 2 min and then raised to 100 °C at 3 °C/min, then from 100 to 180 °C at 5 °C/min, and a total run time of 50.8 min. The volatile compounds analyzed were propan-2-ol, octen-2-ol, hexanal, and nonanal. The mass spectra were obtained using a mass selective detector working in the electronic impact at 70 eV, with a multiplier voltage of 1953 V and collecting data at a rate of 6.34 scans/s over the range, *m*/*z*, 40 to 300. Compounds were identified by comparing their mass spectra with those contained in the NIST05 (National Institute of Standards and Technology, Gaithersburg, Maryland, USA) library. Volatile compounds were quantified as the area percentage under the chromatograme curve.

#### 2.2.3. Microbiological Analysis

Microbiological growth of the total vial count (TVC), total coliform count (TCC), and *Clostridium perfringens* was determined at 50 days from elaboration. Mass seeding was performed on Rapid E. Coli (to determine TCC), PCA (TVC), and Rapid L. mono (*L. monocytogenes*). All media were sterilized at 121 °C for 20 min according to product indications. Peptone water (OXOID, Ltd. CM0087 Basingstoke, Hampshire, United Kingdom) was used to make the dilutions. A laminar flow hood (Telstar, BIO-II-A, Madrid, Spain) was used to carry out the analysis. Finally, plates were incubated for 24 h at 37 °C for TCC, 48 h at 37 °C for TVC, and 48 h at 37 °C for *C. perfringens*. Results were obtained in triplicate and are expressed in cfu/g.

### 2.3. Statistical Analysis

Data were analyzed with the statistical package, SPSS 15.0 (Statistical Package for the Social Science for Window (IBM, Armonk, New York, USA)). The antioxidant and antimicrobial capacity were analyzed using ANOVA. A value of *p* < 0.05 was considered statistically significant. Pearson’s correlation was applied to test differences between groups.

## 3. Results

### 3.1. Characterization of Natural Extracts

#### 3.1.1. Total Phenolic Content (TPC)

The Folin–Ciocalteau method allows a comparative evaluation of the total phenolic content (mg GAE 100 g^−1^) of the diferent extracts. The obtained results are shown in Table 2 and compared with the USDA Database for the Oxygen Radical Absorbance Capacity (ORAC) [23].

As it can be observed, the extract with the highest concentration of phenolic compounds was R, obtained from *Rosmarinus officinalis*, with 1913 mg GAE 100 g^−1^, followed by paprika, C, and oregano, with 1707, 1683, and 1439.7 mg GAE 100 g^−1^, respectively. The natural extracts and ingredients of W, A, Ch, S, and B reported values from 334.7 to 215.3 mg GAE 100 g^−1^, followed by L, garlic, Ce, and Ac, with the lowest quantity of phenolic compounds. A comparison of these results with the obtained values by USDA [23] (Table 2) produces the following order: R > oregano > paprika > S > A > garlic > L > B > Ce. However, there are some differences between both results (determinated in the present study and the USDA database) that could be due to the TPC analysis being carried out directly in fresh vegetables and herbs by USDA, while the results reported in the present study are from food industry by-products or liophilized green leaf vegetables.

#### 3.1.2. Total Nitrate Content (TNC)

Regarding the nitrate content, significant differences (*p* < 0.05) were obtained among the tradicional ingredients from Spanish cuisine and green leaf vegetable extracts (Table 2), whereas natural extracts obtained from citrics, acerola, and rosemary (Ct, Ac, and R) did not report significant results.

As can be observed, leafy green vegetables presented the highest results of nitrates (*p* < 0.05): B, Ch, A, S, Ce, L, and W, followed by oregano, garlic, and paprika, in this order. In addition, in Table 2, these results can be compared with values validated by EFSA [24]. However, A, with 1160.5 ppm NO_3_^−^, showed a great difference regarding the presented values in previous studies. For example, Brkić et al. [25] reported similar results for the nitrate in Ch, S, and L. However, 4354.9 ppm of nitrates were observed in A, similar to the 4677 ppm obtained by EFSA [24]. In the same way, it must be noted that these values were obtained in fresh vegetables, not in lyophilized vegetables, as in the present study. 

On the other hand, oregano presented 51.5 ppm NO_3_^−^, which is a higher value in comparison with the 3 ppm obtained by Dobrinas et al. [26] but it did not present any conclusive result. In the same line, previous results about the nitrate content in paprika were higher than the reported results by EFSA [24] (>100 mg). However, we can contrast with this information by knowing that capsicums and pepper are another important source of nitrates, as shown in Table 2.

#### 3.1.3. Antioxidant Activity

The antioxidant activity of all extracts was measured by four methods, and two of them showed the chelating activity percentages against ABTS (2,2′-azino-bis(3-ethylbenzothiazoline-6-sulphonic acid) and DPPH (2,2-diphenyl-1-picrylhydrazyl) radical cation, in a hydrophilic and lipophilic system, respectively (Table 3). The other two methods showed the efficiency to reduce Fe^3+^ to Fe^2+^ (FRAP) and the hydrophilic antioxidant capacity obtained by measuring the oxygen radical absorbance (ORAC), both expressed in µM Trolox equivalents (TE) 100 g^−1^ (Table 3).

Firstly, considering the results presented in Table 3, it can be reported that beet, acerola, and rosemary showed the highest chelating activity against DPPH and ABTS radical cations. Garlic powder obtained a 51.5% scavenging activity against DPPH, while the lowest value was presented by Ct with 8.45% of quelation power.

On the other hand, the scavenging activity against the hydrophilic radical, ABTS, is generally lower than against DPPH. For this reason, the scavenging activity against ABTS followed the next hierarchy: W, garlic, A, paprika, S, Ch, oregano, Ct, Le, and Ce, with values from 33.4% to 12%, after B, R, and Ac, which presented values of 85.7%, 70.2%, and 46.5%, respectively.

Secondly, in Table 3, we can also observe a similar behaviour regarding the efficiency of each extract to reduce Fe^3+^ to Fe^2+^ by comparing the hydrophilic antioxidant capacity measured by their oxygen radical absorbance. In this case, applying the FRAP method, natural extracts rich in phenolic compounds are the first on the list: R from rosemary, oregano, Ct from citrics, B, W, and paprika followed by Ch, A, L, S, Ac, garlic, and Ce, the last one with 804.7 µM TE 100 g^−1^, 50% less than garlic with 1915.7 or 95% less than R with 17,790 µM TE 100 g^−1^.

Otherwise, the obtained results from the analysis of the oxygen radical absorbance showed the next hierarchy, with similar values published by USDA [23]: R > oregano > paprika > Ct > B > A ≥ Ch ≥ garlic ≥ L ≥ Ac ≥ spinach ≥ watercress > Ce.

#### 3.1.4. Antimicrobial Activity

**Figure 1:**
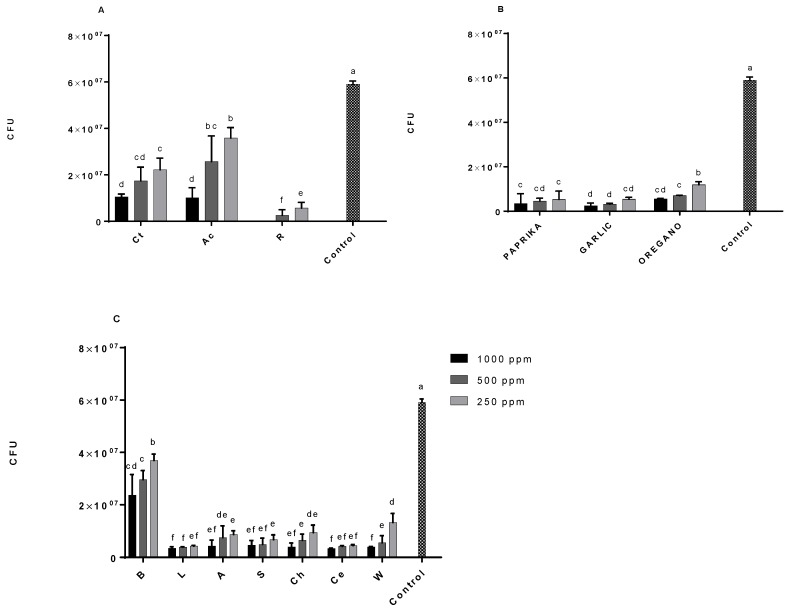
Antimicrobial activity of natural extracts expressed by bacterial growth (cfu) at different concentrations in *Clostridium perfringens* NCTC 8237 CECT 376 after 48 h incubation at 37 °C under anaerobic conditions. (**A**) obtained results for Ct: Citric; R: Rosemary; Ac: Acerola; (**B**) obtained results for Paprika, Garlic, and Oregano; (**C**) obtained results for L: Lettuce; A: Arugula; S: Spinach; Ch: Chard; Ce: Celery; W: Watercress. Superscript letters indicate significant differences (*p* < 0.05) between samples. Control sample represents the normal bacterial growth without any extract.

Figure 1 shows the antimicrobial capacity against *Clostridium perfringens* growth in the presence of all studied extracts, species, and vegetables. In these graphics, it can be appreciated that all extracts reported antimicrobial activity by inhibiting growth or causing bacterial death of *Clostridium perfringens*. 

Taking into account that the control sample represents the total bacterial growth (100% bacteria), it can be observed that B only reduced 65% of bacterial growth, while acerola and C decreased by 85% using 1000 ppm of each extract. Moreover, the rest of the ingredients reduced the bacterial growth between 90% and 100% compared to the control. Similarly, it can be said that the concentration of each ingredient applied directly influences their antimicrobial capacity, at least from 250 to 1000 ppm, because it may be possible that a higher concentration causes a loss of this effect due to saturation of the system. 

On the other hand, the extracts that reported the highest antimicrobial activity (*p* < 0.05) were R (100% at 1000 ppm), followed by garlic, paprika, oregano, and the rest of leafy green vegetables rich in nitrates (Ce, L, S, Ch, A, and W, from 98% to 90%, in this order, at 1000 ppm). Consequently, it can be affirmed that the antimicrobial power of the extracts studied against *Clostridium perfringens* growth is related to the total phenolic and nitrate content. Actually, the bacterial growth (CFU) of *Clostridium perfrigens* has been directly related (*p* < 0.05) to the concentration of nitrates, which was already described by Hasan and Hall [27]. 

In addition, phenolic compounds from R (*Rosmarinus officinalis*) and allicin from garlic have been described as antimicrobial agents acting in different ways: Affecting the cytoplasmic membrane structure, blocking protein synthesis and affecting any of the phases of this process (activation, initiation, binding of the tRNA amino acid complex to ribosomes, or elongation), affecting the metabolism of nucleic acids, and/or blocking any bacterial metabolic pathways.

### 3.2. Volatile Compounds and Microbiological Spoilage in Spanish Chorizo

Once the antioxidant and antimicrobial capacities of each ingredient were measured in vitro, they were incorporated as preservative agents to retard the lipid oxidation and the microbiological growth in a cured meat product.

#### 3.2.1. Volatile Compounds

The obtained results of volatile fatty acids analysis by GS-MS are shown in Table 4.

Volatile compounds from lipid oxidation (propan-2-ol, hexanal, and nonanal) were significantly affected (*p* < 0.05) by the ripening time and addition of antioxidants (Table 3). In contrast, octen-2-ol was not affected by the ripening time or addition of antioxidants.

In general, 2-propanol increased from 0.45 to 1.75 mg/g meat during 125 days area units to 316 × 106 area units during the first 4 days. In contrast, the increase in samples with natural extracts was less pronounced, especially R_LAW_ with a value of 0.85 mg/g at day 150. The production of octen-2-ol was not detected in any of the samples, suggesting good product sensory quality because these compounds have a low threshold off-odour. 

The behaviour of nonanal and hexanal was quite similar, with both increasing during storage and showing significant differences between the control and samples with extracts from day 50 onwards. Nonanal is associated with waxy and painty descriptors, while 1-octen-3-ol is amongst the compounds responsible for rancid odours and it is an autoxidation indicator of linoleic and arachidonic acids. In addition, hexanal is an aldehyde that can be generated from arachidonic acids, oleic acid, and through the degradation of deca-2,4-dienal [28].

Volatile alcohols, such as heptanol, are formed from oleic acid [29], whereas pentanol and 1-octen-3-ol are by-products of the autoxidation of linoleic and arachidonic acids [30].

Hexanal concentration ranging from 2 to 7 g kg^−1^ was reported in cooked pork [31] cooked turkey [32] and cooked ground beef [33,34].

The addition of antioxidants decreased the total volatile compounds from lipid oxidation (2-propanol, hexanal, and nonanal). At the end of process, hexanal contents were found in the following order: C, R_LAW_, R_SCe_, C_LAW_, R_ChB_, C_SCe_, and C_ChB_ and their mean values were 0.44, 0.18, 0.19, 0.19, 0.20, 0.21, and 0.25 mg/g, respectively. These results indicated that the addition of R and Ct improved the control of lipid oxidation compared to the control sample. These results are consistent with the polyphenol content and the in vitro evaluation of the antioxidant activity of the extracts (Table 3).

According to Kerler and Grosch [28], all volatile compounds analysed (hexanal, heptanal, octen-2-ol, and propan-2-ol) are components that contribute the most to the emergence of unpleasant notes of flavour due to their high rate of formation and low flavour threshold [35].

The loss of acceptance depends to a large extent on odour and flavour deterioration in meat and meat products [36]. In general, the volatile profile of chorizo strongly depends on the composition. Polyphenols are metal chelating agents and also act on free radicals, since their benzene rings inhibit chain reactions during lipid oxidation. Previous studies have demonstrated that a rosemary diet delays lipid oxidation in raw meat from broilers [37], pigs [38], and lambs [39].

#### 3.2.2. Microbiological Content

The antimicrobial capacity of different extracts was studied in cured meat products elaborated with pork meat. Consequently, the microbiological results of Spanish chorizo after 50 days from elaboration are shown in Table 5.

As can be appreciated, the only sample that presented *Clostridium perfringens* growth was the control sample, while the rest of samples enriched with natural extracts (R_LAW_, R_SCe_, R_ChB_ C_LAW_, C_SCe_, and C_ChB_) presented an absence of this bacteria in the 10 g sample. Similarly, samples that incorporated R or Ct extracts in their formula decreased from 24% to 60% of the total coliform count in all the samples compared to the control. This fact could be due to the presence of monoterpens and rosmarinic acid from the R extract in case of R_LAW_, R_SCe_, and R_ChB_, or the presence of hesperidin in the case of C_LAW_, C_SCe_, and C_ChB._ It is also important to note that the combination of L, A, and W with R was more effective than combined with Ct, while the mix among S and Ce with Ct presented lower bacterial growth than with R. 

Otherwise, the total viable bacteria growth was lowest in samples enriched with citric extract (C_LAW_, C_SCe_, and C_ChB_), which demonstrated the synergism between Ct and natural nitrate sources. This behaviour was not visible after the combination of R and natural nitrate sources. This synergistic activity could be due to the reaction between the flavonoid, hesperidin, with other phenolic compounds from L, A, W, S, Ce, Ch, and B, such as flavonoids quercetin, kaempferol, and apigenin, or phenolic acids, such as gallic, ferulic, caffeic, and *p*-coumaric acid. In addition, this reaction could also be produced among nitrates and hesperidin. 

## 4. Discussion

Actually, all these extracts are obtained from natural foods, fruits, vegetables, and herbs, known to be excellent sources of phenolic compounds. For example, R is a natural rosemary (*Rosmarinus officinalis*) extract, with hydrophobic powder containing 14.6% carnosic acid and 5.8% carnosol, which justifies the TPC result shown. In the same way, C, the citric extract, contained 55.11% flavonoids as hesperidin measured by HPLC. On the other hand, paprika was shown to have a high concentration of capsaicin, a phenolic compound responsible for its characteristic colour and flavour [40]. For example, Škrovánková et al. [7] announced similar results for the TPC in different paprika spices, from 1467 to 2878 mg GAE 100 g^−1^, while the USDA database reported 1643 mg GAE 100 g^−1^ in the same ingredient. Similarly, Kruma et al. [41] obtained from 72.12 to 52.15 mg phenolic compounds per 100 g of oregano using different solvents for the extraction, with methanol or ethanol. However, the USDA database described a 3789 mg GAE 100 g^−1^ product. Considering that different phenolic compounds have been indentified in this herb, such as phenolic acids and its derivatives (caffeic, rosmarinic acid, and their dimmers), flavons (apigenin and luteolin), and flavonols (eriodictyol or naringenin), the obtained result can also be justified [42].

Regarding the green leaf vegetables analyzed, W, A, Ch, S, L, B, and Ce, previous studies showed comparative TPC values. For example, Zeb [43] reported 290 mg phenolic compounds per 100 g of watersoluble extract of watercress roots. Corleto et al. [44] showed 600 µg GAE/mL beetroot juice and 780 µg GAE/mL arugula juice. Alarcón-Flores et al. [45] described 70 mg phenolic compounds per kg of spinach; Pyo et al. [46] reported 290 mg GAE 100 g^−1^ in chard. Pérez-López et al. [47] obtained 100 mg GAE g^−1^ in lettuce, while Yao et al. [48] reported lower values in celery, from 3.48 to 5.02 mg GAE 100 g^−1^. This fact can be explained by the concentration of flavonoids, such as catechins, myricetin, quercetin, and kaempferol, or phenolic acids, such as gallic, *p*-hydroxybenzoic, protocatechuic, syringic, vanilic, chlorogenic, caffeic, *p*-coumaric, or ferulic acid, which have been described in all references previously cited.

Finally, garlic and Ac were reported to have lower TPC values due to these extracts containing higher quantities of allicin or vitamin C, respectively. However, garlic has also shown phenol structures in its formula, such as phenolic acids (caffeic and ferulic acid) and flavonoids (apigenin and quercetin) [45]. While Vendramini and Trugo [49] reported that the content of anthocyanins or ripe acerola skin was estimed as 37.5 mg per 100 g.

It can be interpreted tthat the scavenging power of different extracts lies in their composition and the molecular structure of bioactive substances, such as the presence of catechol and gallate groups in phenol groups, their polymerization and conjugation, or the combination with other substances, such as nitrates, pigments, and/or vitamins.

In this way, R is a natural extract obtained from *Rosmarinus officinalis* L. with 14.59% carnosic acid, 5.84% carnosol, and 0.60% 12-O-methylcarnosic acid, while C obtained from *Citrus sinensis* L. contains 55.11% hesperidin as has been described previously. Considering this, it can be understood why the highest values in the FRAP and ORAC analysis were obtained by R. However, it must be noted that the antioxidant behaviour of flavanones (C) varies according to the oxidant radical used. For example, Gardner et al. [50] reported that the antioxidant power of flavanones obtained by DPPH* was much lower than that using ABTS*, which was also proven in the present study. Moreover, the DPPH* results are less sensitive to hydrophilic antioxidants [51]. This aspect could explain the lower values obtained by measuring ABTS and DPPH radical scavenging activity, in comparison with the high antioxidant activity shown by the FRAP and ORAC method, which showed higher values that are justified by the high hesperedin content.

On the other hand, the Ac extract from *Malpighia emarginata*, with 5% vitamin C, is also rich in phenolic compounds, such as anthocyanins, anthocyanidin, phenolic acids (*p*-coumaric, caffeic, and ferulic acid), flavonols (quercetin and kaempferol), and catechins [6,49]. In addition, it contains β-carotene and minerals [52], which make it a functional fruit and justifies the results obtained from the different analyses carried out in the present study.

The traditional ingredients from Spanish cuisine also had higher values in the antioxidant assays but behaved differently according to the method used for assessment. For instance, the antioxidant activity of oregano, paprika, and garlic was higher when measured by the FRAP method and ORAC method. The antioxidant activity of oregano is mainly due to the concentration of phenol and catechol groups in the molecular structure of its principal phenolic compounds, such as oreganoside. On the other hand, paprika is a source of important compounds for its antioxidant capacity, such as carotenoids, capsaicinoids, and vitamins C and E (α and γ-tocopherol from pepper seeds) [53]. However, the concentration of this kind of compound varies due to several reasons, like the crop, the degree of ripeness, or the temperature used to air-dry the peppers [54]. This fact can explain the differences among the values published by USDA [23] and those obtained in the present study. 

Additionally, garlic is an excellent scavenger of hydroxyl radicals due to its content of flavonoids (quercetin and kaempferol) and organosulphurs (allyl-cysteine, dialyl sulphide, and dialyl trisulphide) [1]. Thiosulphonated compounds, such as allicin, provide the characteristic odour to garlic, however, this compound is related to its anti-iflammatory activity but not with its antioxidant effect [55], which also can explain the obtained results in the present study.

Finally, the obtained results regarding leafy green vegetables can also be related to their concentrations in phenolic compounds. For example, B, with the highest scavenging power against DPPH and ABTS radicals, is the principal source of nitrates, as was comented previously, and it is also rich in phenolic compounds, with 215.3 mg GAE 100 g^−1^, like anthocyanidins. In addition, the natural purple colour of beet is due to the presence of betanin, also known for its antioxidant power, being a derivative from betalamic acid, which is obtained from the L-DOPA molecule. Moreover, different authors have obtained comparative results, such as Saani and Lawrence [56], who showed a 50% scavenging DPPH radical activity, or Ou et al. [57], who obtained a higher antioxidant capacity using ORAC and FRAP assays in beet of 11500 and 8600 µM TE 100 g^−1^, respectively. However, the USDA [23] published a lower result more similar to that obtained in the present study of 1946 µM TE 100 g^−1^. 

The remaining leafy green vegetables obtained similar results in the different antioxidant assays, which can be associated with the fact that they also share the same bioactive compounds: Phenolic acids (gallic, ferulic, caffeic, and *p*-coumaric acids), flavonoids (quercetin, kaempferol, and apigenin), and chlorophyll as the principal pigment responsible for their green colour. In the same way, it can also be appreciated that celery had a lower antioxidant capacity than other vegetables. However, this may be related to the lower phenolic content, also described previously, and the similar result shown by USDA [23].

Regarding the obtained results of oxidative damage of Spanish chorizo for 125 days, acids, which were practically absent at the start, showed the largest increase among the volatiles during ripening. Carbohydrate metabolism [58], lipolysis [59], amino acid catabolism [60], and smoke [61] might account for the formation of these acids. The only alcohol present in all the samples of chorizo was furfurylalcohol. Johansson et al. [62] reported the presence of this compound as the major alcohol in a smoked dry fermented sausage. Lipid oxidation [63,64,65], carbohydrate metabolism [58], and amino acid catabolism [60] could be the most important pathways accounting for the production of volatile alcohols in fermented dry sausages. These compounds could also come from smoke, like furfurylalcohol, which is abundant in wood smoke [61,66]. 

A total lack of straight chain aldehydes and ketones, which are typical breakdown products of the hydroperoxides derived from fatty acids [63], was observed in chorizo unlike other varieties of dry fermented sausage [67]. On the other hand, branched and cyclic carbonyls were detected in greater profusion. The bulk of the carbonyls was formed during ripening, and each of the ketones isolated increased during ripening, whereas the aldehydes did not show a definite evolution. The presence of some cyclo-pentanones and cyclopentanones as volatile constituents of dry fermented sausages has not been previously reported. Nonetheless, these substances are typical of wood smoke [61,66]. The presence of methyl-branched aldehydes may be explained by amino acid catabolism [60] and by ketones (such as diacetyl, acetoin) and hydroxypropanone by carbohydrate metabolism [58]. Large amounts of furfural and 5-methylfuran-2-carbaldehyde, which are characteristic products of the Maillard reaction, were observed principally in industrial chorizo. Apart from spices and smoke, it is generally accepted that the formation of volatiles during the ripening of dry fermented sausages would be due to the occurrence of a set of reactions between the precursors of flavor, such as carbohydrates, lipids, and proteins, with microbial or endogenous enzymes being involved in many instances. Several low molecular weight compounds isolated from chorizo, i.e., formic, acetic, and propanoic acids, propanol, butan-2,3-diol, diacetyl, 1-hydroxy-2-propanone, acetoin, ethyl acetate, ethyl propionate, propyl acetate, and ethyl butyrate, might derive to a great extent, whether directly or indirectly, from carbohydrate metabolism [58]. There was approximately twice the quantity of these substances in industrial chorizo with regard to the traditional ones. Therefore, a more intense fermentation metabolism in industrial chorizo seemed probable. The production of 2-methylpropanal, 2- and 3-methylbutanal, 2-methylpropanol, 2- and 3-methylbutanol, 2-methylpropanoic, and 2- and 3-methylbutanoic acids from valine, leucine, and isoleucine would be explained by amino acid degradation [60]. The larger total content of these substances in industrial chorizo would imply that major amino acid catabolism developed in this type of chorizo. On the other hand, it appeared that there was a smaller incidence of amino acid degradation as contrasted with carbohydrate fermentation in chorizo. This fact was also reported by Berdague et al. [68].

Lipid autooxidation accounts for the appearance of numerous volatile compounds in dry fermented sausage [69,70]. However, the absence of key intermediates of autooxidation in the chorizo analyzed implies that the development of lipid oxidation is irrelevant, aromatically speaking. This was also suggested by Berger et al. [71] for another type of dry sausage. This could be due to the antioxidant effect of paprika and smoke. The addition of curing agents, which possess a positively recognised antioxidant effect, seemed to produce no especially marked repercussions on the flavour of chorizo in light of the following two points. First, it was not possible to impute to the curing agents a restrictive effect on the formation of volatiles originating from chemical oxidation, since these substances were not observed either in industrial or in traditional chorizo.

In parallel, the obtained results from the antimicrobial in vitro test of each ingredient and natural extract showed that natural nitrate sources and rosemary especially presented an excellent antimicrobial activity against *Clostridium perfringens.* This fact is due to the presence of nitrates that directly affect bacterial growth, as was also described by Hasan and Hall [29]. Nevertheless, when these combined extracts were used in cured meat products, such as Spanish chorizo in the present study, Ct combined with natural nitrate sources showed a synergistic activity that was not shown by R in the same conditions. This behaviour must be studied in future research, but now it can be affirmed that the reaction among flavonoids as hesperidin and natural nitrate sources from green leafy vegetables demonstrates a synergistic effect in the preservation of cured meat products, which is also elaborated by paprika, oregano, garlic, and acerola extract, which is also rich in Vitamin C. Furthermore, they are able to increase the antioxidant and antimicrobial activity of the studied ingredients by separation. This increas could be produced by vitamin C, which acts as a proton donor to the phenolic compounds, whose hydroxyl groups are responsible for the antioxidant and antimicrobial capacity.

These reactions can explain the antimicrobial activity that can be produced by different methods. For instance, by affecting the cytoplasmic membrane structure, blocking protein synthesis, affecting any of the phases of this process (activation, initiation, binding of the tRNA amino acid complex to ribosomes, or elongation), affecting the metabolism of nucleic acids, and/or blocking any bacterial metabolic pathways.

## 5. Conclusions

Rosemary was the most antimicrobial compound followed by nitrate natural sources and spices, such as paprika, garlic, and oregano, which can be compared with their antioxidant capacity. However, citrus extract was the only compound that presented a higher antioxidant capacity, due to its hesperedin content, but a lower antimicrobial capacity. Nevertheless, the combination of citric extract with leafy green vegetables extracts rich in nitrates showed a higher antimicrobial activity regarding the rosemary and the control sample. This fact demonstrates the synergism among the flavonoid hesperidin and natural nitrate sources, but this behaviour did not present the same effectiveness in combination with the monoterpenes from rosemary extracts (carnosic acid and carnosol). The combination of citrus and rosemary extract in cured meat products, such as chorizo, is a good alternative in order to produce clean label meat product free of synthetic additives.

## Figures and Tables

**Table 1:** Formulation of Spanish chorizo samples.

	Samples						
Ingredients	Control	C_LAW_	C_SCe_	C_ChB_	R_LAW_	R_SCe_	R_ChB_
Pork meat (g)	875	875	875	875	875	875	875
Pork fat (g)	1350	1350	1350	1350	1350	1350	1350
Water (mL)	75	75	75	75	75	75	75
Commercial mix^®^ (g/kg)	65						
Paprika (g/kg)		30	30	30	30	30	30
Oregano (g/kg)		3	3	3	3	3	3
Garlic (g/kg)		3	3	3	3	3	3
Dextrose (g/kg)		3	3	3	3	3	3
Salt (g/kg)		5	5	5	5	5	5
Meat protein (g/kg)		23	23	23	23	23	23
Ferment (mL)	20	20	20	20	20	20	20
Natural extracts (ppm):							
Ct		200	200	200			
R					200	200	200
Ac		100	100	100	100	100	100
LAW		3000 + 1500 + 1500			3000 + 1500 + 1500		
SCe			3000 + 3000			3000 + 3000	
ChB				3000 + 3000			3000 + 3000

Commercial mix^®^: composed of spices, salt, dextrose, lactose, milk protein, emulsifiers (triphosphates E-451, diphosphate E-450), flavour enhancer (monosodium glutamate E-621), preservative (sodium nitrate E-251), antioxidant (sodium ascorbate E-301), and colouring (carminic acid E-120). Ct: Citric; R: Rosemary; Ac: Acerola; LAW: Lettuce + arugula + watercress; SCe: Spinach + celery; ChB: Chard + beet.

**Table 2:** Total phenolic content (TPC) (mg GAE 100 g^−1^) and total nitrate content (TNC) (ppm NO_3_^−^) in natural extracts (M ± SD), together with their comparison with USDA [23] and EFSA [24] results.

Samples	Total Phenolic Content		Total Nitrate Content	
	mg GAE 100 g^−1^	mg GAE 100 g^−1^ [23]	ppm NO_3_^−^	ppm NO_3_^−^ [24]
Ct	1683.7 ± 8.6 ^c^	Nd	Nd	Nd
Ac	57.67 ± 1.5 ^i^	Nd	Nd	Nd
R	1913 ± 29 ^a^	4980	Nd	Nd
Paprika	1707 ± 20.1 ^b^	1643	21.8 ± 0.5 ^i^	108
Garlic	87.3 ± 2.5 ^hi^	92	50.2 ± 0.7 ^h^	69
Oregano	1439.7 ± 7.5 ^d^	3789	51.5 ± 0.3 ^h^	Nd
B	215.3 ± 9.6 ^ef^	55 *	1384.1 ± 1.2 ^a^	1852 *
L	145.3 ± 5.1 ^fg^	90 *	736.4 ± 0.9 ^f^	1324 *
A	296.3 ± 5.7 ^ef^	125 *	1160.5 ± 1.0 ^c^	4677 *
S	255 ± 6 ^ef^	205 *	948.8 ± 0.8 ^d^	1066 *
Ch	278 ± 37 ^ef^	Nd	1213.4 ± 1.5 ^b^	1690 *
Ce	80 ± 1 ^hi^	42 *	921.3 ± 1.1 ^e^	1103 *
W	334.7 ± 4 ^e^	Nd	472.9 ± 0.8 ^g^	136 *

Ct: Citric; R: Rosemary; Ac: Acerola; L: Lettuce; A: Arugula; S: Spinach; Ch: Chard; Ce: Celery; W: Watercress. Superscript letters indicate significant differences (*p* < 0.05) between samples. M ± SD: Mean ± standard deviation. * These data correspond to fresh vegetables. Nd: no data found.

**Table 3:** Antioxidant activity of natural extracts by measuring their ABTS, and DPPH radical scavenging activity, together with their ORAC and FRAP (µM TE g^−1^) (M ± SD).

Samples	Chelating Activity Percent (%)		Antioxidant Activity (µM TE g^−1^ ± SD)		
	ABTS	DPPH	ORAC	FRAP	ORAC [23]
Ct	15.4 ± 0.2 ^h^	8.45 ± 0.3 ^k^	4828.5 ± 19.9 ^d^	6004.7 ± 29.6 ^c^	Nd
Ac	46.5 ± 0.3 ^c^	78.3 ± 0.5 ^b^	16,80.7 ± 19.3 ^g^	1925.7 ± 28.7 ^f^	Nd
R	70.2 ± 0.1 ^b^	76.7 ± 1.7 ^c^	19,909.0 ± 59.8 ^a^	17,790 ± 53.3 ^a^	112,200 **
Paprika	21.1 ± 1.6 ^f^	48.7 ± 0.2 ^ef^	5746.0 ± 21.7 ^c^	2491.3 ± 17.1 ^e^	13,750
Garlic	25.4 ± 0.8 ^e^	51.5 ± 0.3 ^d^	1919.3 ± 23.4 ^g^	1915.7 ± 52.5 ^f^	450
Oregano	15.6 ± 0.5 ^h^	41.3 ± 0.2 ^j^	11,436.7 ± 27.5 ^b^	9355.3 ± 46.4 ^b^	13,970
B	85.7 ± 1.1 ^a^	90.2 ± 0.6 ^a^	3509.0 ± 26.3 ^e^	3690 ± 58.8 ^d^	1946 *
L	14.6 ± 1.1 ^i^	49.9 ± 0.1 ^e^	1723.3 ± 35.1 ^g^	1998 ± 18.9 ^f^	1321 *
A	25.9 ± 3.1 ^e^	49.2 ± 1.2 ^e^	2881.3 ± 28.4 ^f^	2071 ± 16.3 ^ef^	1904 *
S	20.1 ± 0.1 ^g^	43.6 ± 3.6 ^i^	1491.3 ± 22.1 ^gh^	1995.3 ± 9.6 ^f^	1513 *
Ch	19.7 ± 0.0 ^g^	47.4 ± 0.6 ^g^	2150.7 ± 35.0 ^fg^	2216.7 ± 19.4 ^e^	Nd
Ce	12.0 ± 0.5 ^j^	48.7 ± 0.4 ^ef^	993.7 ± 18.5 ^i^	804.7 ± 33.6 ^g^	512 *
W	33.4 ± 2.6 ^d^	46.5 ± 0.1 ^h^	1200.7 ± 15.0 ^h^	2510.3 ± 39.4 ^e^	Nd

Ct: Citric; R: Rosemary; Ac: Acerola; L: Lettuce; A: Arugula; S: Spinach; Ch: Chard; Ce: Celery; W: Watercress. Superscript letters indicate significant differences (*p* < 0.05) between natural extracts. M ± SD: Mean ± standard deviation; TE: Trolox equivalents. * These data correspond to fresh vegetables. ** This data corresponds to dried rosemary. Nd: no data found.

**Table 4:** Average values and standard deviations of volatile compounds (mg/g meat) in chorizo stored for 0, 25, 50, and 125 days, under retail conditions.

Volatile Compounds	Sample	Day 0	Day 25	Day 50	Day 125
propan-2-ol	Control	0.45 ± 0.02	0.54 ± 0.02	1.02 ± 0.01 ^a^	1.75 ± 0.03 ^a^
	R_LAW_	0.37 ± 0.01	0.46 ± 0.01	0.37 ± 0.02 ^b^	0.85 ± 0.04 ^c^
	R_SCe_	0.38 ± 0.03	0.44 ± 0.02	0.92 ± 0.01 ^b^	1.10 ± 0.05 ^b^
	R_ChB_	0.58 ± 0.02	0.65 ± 0.01	0.66 ± 0.02 ^b^	1.27 ± 0.01 ^b^
	C_LAW_	0.65 ± 0.01	0.70 ± 0.01	0.89 ± 0.03 ^b^	1.20 ± 0.02 ^b^
	C_SCe_	0.61 ± 0.03	0.69 ± 0.03	0.59 ± 0.01 ^b^	1.33 ± 0.00 ^b^
	C_ChB_	0.38 ± 0.02	0.50 ± 0.02	0.55 ± 0.01 ^b^	1.08 ± 0.01 ^b^
octen-2-ol	Control	0.11 ± 0.01	0.10 ± 0.00	0.15 ± 0.01	0.10 ± 0.01
	R_LAW_	0.10 ± 0.02	0.18 ± 0.02	0.15 ± 0.02	0.15 ± 0.02
	R_SCe_	0.14 ± 0.02	0.18 ± 0.01	0.12 ± 0.01	0.11 ± 0.01
	R_ChB_	0.14 ± 0.01	0.13 ± 0.01	0.16 ± 0.02	0.25 ± 0.02
	C_LAW_	0.12 ± 0.01	0.12 ± 0.02	0.14 ± 0.03	0.19 ± 0.01
	C_SCe_	0.16 ± 0.00	0.15 ± 0.01	0.15 ± 0.01	0.16 ± 0.02
	C_ChB_	0.10 ± 0.01	0.13 ± 0.01	0.13 ± 0.01	0.11 ± 0.01
Hexanal	Control	0.11 ± 0.01	0.14 ± 0.02	0.21 ± 0.02 ^a^	0.44 ± 0.03 ^a^
	R_LAW_	0.12 ± 0.01	0.14 ± 0.01	0.08 ± 0.01 ^b^	0.18 ± 0.01 ^b^
	R_SCe_	0.10 ± 0.02	0.12 ± 0.01	0.12 ± 0.03 ^b^	0.18 ± 0.02 ^b^
	R_ChB_	0.13 ± 0.01	0.16 ± 0.00	0.15 ± 0.02 ^b^	0.20 ± 0.01 ^b^
	C_LAW_	0.11 ± 0.02	0.14 ± 0.02	0.09 ± 0.00 ^b^	0.19 ± 0.02 ^b^
	C_SCe_	0.12 ± 0.01	0.14 ± 0.01	0.18 ± 0.01 ^b^	0.21 ± 0.01 ^b^
	C_ChB_	0.13 ± 0.03	0.12 ± 0.01	0.19 ± 0.01 ^b^	0.25 ± 0.02 ^b^
Nonanal	Control	0.18 ± 0.01	0.39 ± 0.04	0.45 ± 0.02 ^a^	0.58 ± 0.01 ^a^
	R_LAW_	0.17 ± 0.01	0.27 ± 0.03	0.32 ± 0.01 ^b^	0.41 ± 0.03 ^b^
	R_SCe_	0.22 ± 0.01	0.16 ± 0.01	0.27 ± 0.02 ^b^	0.27 ± 0.02 ^b^
	R_ChB_	0.14 ± 0.01	0.18 ± 0.01	0.35 ± 0.01 ^b^	0.30 ± 0.02 ^b^
	C_LAW_	0.15 ± 0.02	0.18 ± 0.02	0.20 ± 0.03 ^b^	0.23 ± 0.01 ^b^
	C_SCe_	0.18 ± 0.03	0.15 ± 0.01	0.27 ± 0.02 ^b^	0.24 ± 0.02 ^b^
	C_ChB_	0.17 ± 0.02	0.10 ± 0.01	0.19 ± 0.01 ^b^	0.25 ± 0.01 ^b^

R_LAW_: 500 ppm rosemary extract + 250 ppm acerola + 3000 ppm lettuce, arugula, and watercress; R_SCe_: 500 ppm rosemary extract + 250 ppm acerola + 3000 ppm spinach and celery; R_ChB_: 500 ppm rosemary extract + 250 ppm acerola + 3000 ppm chard and beet; C_LAW_: 500 ppm citric extract + 250 ppm acerola + 3000 ppm lettuce, arugula, and watercress; C_SCe_: 500 ppm citric extract + 250 ppm acerola + 3000 ppm spinach and celery; C_ChB_: 500 ppm citric extract + 250 ppm acerola + 3000 ppm chard and beet. Superscript letters indicate significant differences (*p* < 0.05) between natural extracts.

**Table 5:** Microbiological results (cfu/g) of Spanish chorizo analysis after 50 days under refrigerated storage.

Samples	Analysis		
	TVC	TCC	*Clostridium perfringens*
Control	6.20 × 10^4 b^	2.77 × 10^2^	10 ^a^
R_LAW_	5.12 × 10^5 a^	1.28 × 10^2^	Absence in 10 g ^b^
R_SCe_	4.25 × 10^5 a^	2.01 × 10^2^	Absence in 10 g ^b^
R_ChB_	3.62 × 10^5 a^	1.10 × 10^2^	Absence in 10 g ^b^
C_LAW_	4.05 × 10^4 b^	1.56 × 10^2^	Absence in 10 g ^b^
C_SCe_	6.22 × 10^4 b^	1.79 × 10^2^	Absence in 10 g ^b^
C_ChB_	5.98 × 10^4 b^	2.10 × 10^2^	Absence in 10 g ^b^

RLAW: 500 ppm rosemary extract + 250 ppm acerola + 3000 ppm lettuce, arugula, and watercress; RSCe: 500 ppm rosemary extract + 250 ppm acerola + 3000 ppm spinach and celery; RChB: 500 ppm rosemary extract + 250 ppm acerola + 3000 ppm chard and beet; CLAW: 500 ppm citric extract + 250 ppm acerola + 3000 ppm lettuce, arugula, and watercress; CSCe: 500 ppm citric extract + 250 ppm acerola + 3000 ppm spinach and celery; CChB: 500 ppm citric extract + 250 ppm acerola + 3000 ppm chard and beet. Superscript letters indicate significant differences (*p* < 0.05) between natural extracts. TVC: Total viable count; TCC: Total coliform count.
